# 1-(Methyl-α-d-gluco­pyran­osid-6-yl)-3-vinyl­imidazolium iodide di­methyl­formamide monosolvate

**DOI:** 10.1107/S2414314622002656

**Published:** 2022-03-10

**Authors:** Sina Lambrecht, Alexander Villinger, Stefan Jopp

**Affiliations:** a Department Life, Light & Matter, University of Rostock, Albert-Einstein-Str. 25, 18059 Rostock, Germany; b Institute of Chemistry, University of Rostock, Albert-Einstein-Str. 3a, 18059 Rostock, Germany; Katholieke Universiteit Leuven, Belgium

**Keywords:** crystal structure, carbohydrate, imidazolium

## Abstract

The title solvated molecular salt, [MeGluVIm]I (MeGluVIm = 1-(methyl-α-d-gluco­pyran­osid-6-yl)-3-vinyl­imidazolium), or C_12_H_19_N_2_O_5_^+^·I^−^·C_3_H_7_NO, was synthesized from methyl-α-d-6-iodo­gluco­pyran­oside and vinyl­imidazole in DMF. It crystallizes through precipitation from ethyl acetate solution directly after the reaction procedure. The crystal structure consists of an iodide anion and a [MeGluVIm] cation. Furthermore, the crystal structure contains one mol­ecule of DMF, which accepts two O—H⋯H hydrogen bonds from the OH groups of the gluco­pyran­oside.

## Abstract 2

The title compound is a gluco­pyran­oside compound containing a cationic vinyl­imidazolium moiety. The gluco­pyran­oside ring shows a distinctive chair conformation.

## Graphical abstract

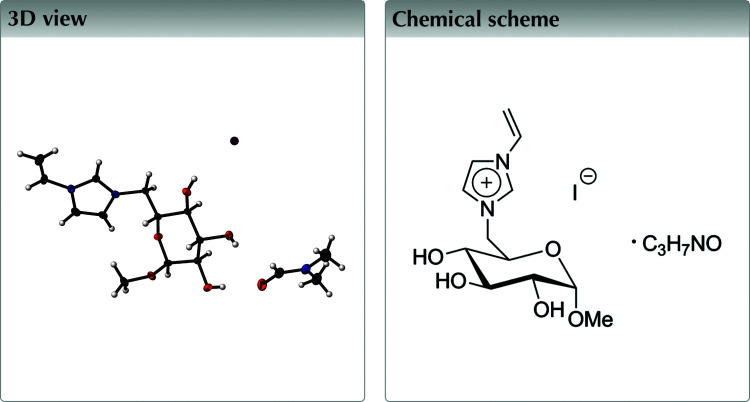

## Structure description

**Figure 1:**
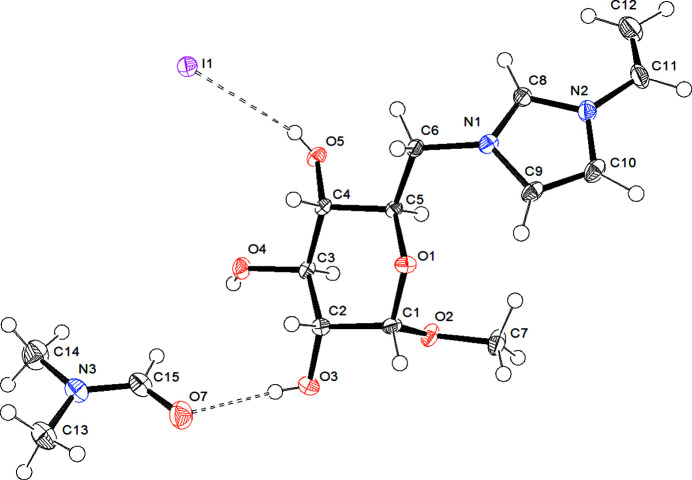
Mol­ecular structure of the title compound. Displacement ellipsoids correspond to 50% probability.

**Figure 2:**
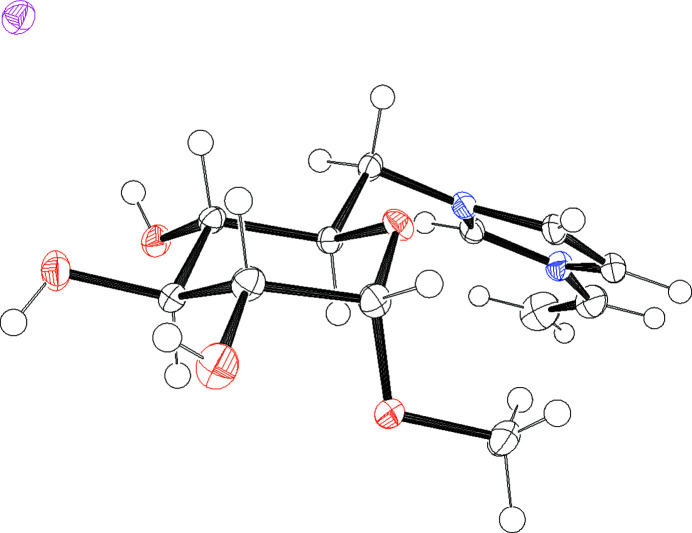
Mol­ecular structure of the title compound. Displacement ellipsoids correspond to 50% probability. The DMF was removed for a clear view of the chair conformation.

[MeGluVIm]I is part of a sub-category of ionic liquids, called carbohydrate-based ionic liquids (CHILs; Jopp, 2020 ▸). These mol­ecules are defined as ionic organic compounds in which either the cation or the anion consists of an intact carbohydrate moiety. Our group has recently discovered a straightforward synthetic strategy for CHILs, in which methyl-α-d-gluco­pyran­oside is transformed into methyl-α-d-6-iodo­gluco­pyran­oside in the first step (Skaanderup *et al.*, 2002 ▸) and then in the second step quarternized with an *N*-substituted imidazole of choice to achieve a carbohydrate-based ionic liquid (Schnegas & Jopp, 2021 ▸). The title compound [MeGluVIm]I contains a vinyl­imidazolium ring bound to atom C6 of the gluco­pyran­oside. Fig. 1 ▸ shows the asymmetric unit, including one mol­ecule of di­methyl­formamide, which was used as the reaction solvent. The title compound crystallizes in a monoclinic unit cell. The crystal structure contains three classical hydrogen bonds and additional C—H⋯O/I inter­actions (Table 1 ▸). One hydrogen bond is formed between O3—H3*A* of the gluco­pyran­oside and O7 of DMF with an H⋯H length of 2.09 (4) Å. Two additional hydrogen bonds exists between the [MeGluVIm] cation and the iodide anion, which are O4—H4*A*⋯I1 with 2.71 (5) Å and O5—H5*A*⋯I1 with 2.75 (5) Å. Fig. 2 ▸ gives an alternative view of the cation, indicating the distinctive chair conformation of the gluco­pyran­oside as well as the overall stereochemistry of the compound. The configurations of the stereogenic centres in the chosen cation are *S* (C1), *R* (C2), *S* (C3), *S* (C4) and *R* (C5).

## Synthesis and crystallization

Methyl-6-iodo-α-d-gluco­pyran­oside (1.824 g; 6 mmol) and 1-vinyl­imidazole (0.821 g; 10 mmol) were dissolved in DMF (10 ml) and stirred at 95°C for 24 h. After cooling down, ethyl acetate (80 ml) was added and the flask was stored in a fridge overnight. The solvent was deca­nted and the precipitated solid was washed with ethyl acetate (3 × 40 ml) and dried under high vacuum to achieve the product as a beige solid (1.752 g; yield 73%). Single crystals of the compound were formed during the precipitation (m.p.: 448–453 K; *T_d_*: 509 K).

^1^H NMR (300 MHz, D_2_O): δ = 3.21–3.30 (*m*, 3H, OCH_3_); 3.58 (*dd*, 1H, ^3^ *J* = 9.77, ^3^ *J* = 3.77, H-2); 3.66–3.75 (*m*, 1H); 3.95 (*dd*, 1H, ^3^ *J* = 6.3, ^3^ *J* = 3.72); 4.50 (*dd*, 1H, ^3^ *J* = 14.55, ^3^ *J* = 7.38, H-6a); 4.70 (*dd*, 1H, ^3^ *J* = 14.55, ^3^ *J* = 2.55, H-6 b); 4.85 (*d*, 1H, ^3^ *J* = 3.77, H-1); 5.49 (*dd*, 1H, ^3^ *J* = 8.68, ^3^J = 2.84, vinyl-CH); 5.86 (*dd*, 1H, ^3^ *J* = 15.58, ^3^ *J* = 2.85, vinyl-CH_2 − a_); 7.2 (*dd*, 1H, ^3^ *J* = 15.58, ^3^ *J* = 8.70, vinyl-CH_2 − b_); 7.70 (*d*, 1H, ^3^J = 2.0, H_Ar_); 7.86 (*d*, 1H, ^3^ *J* = 2.0, H_Ar_); 9.16 (*s*, 1H).

^13^C NMR (300 MHz, D_2_O): δm= 36.9 (NCH); 50.2 (C-6); 55.1 (OCH_3_); 69.2, 40.5, 71.0, 72.8 (C-2, C-3, C-4, C-5); 99.3 (C-1); 109.8 (CH_2_); 119.4, 123.8, 128.1 (CH_Ar_).

HRMS (ESI, *m*/*z*): calculated for C_12_H_19_N_2_O_5_^+^, 271.1299; measured 271.1306. Calculated for I^−^, 126.9040; measured 126.9045.

## Refinement

Crystal data, data collection and structure refinement details are summarized in Table 2 ▸. The crystal studied was refined as a two-component inversion twin.

## Supplementary Material

Crystal structure: contains datablock(s) I. DOI: 10.1107/S2414314622002656/vm4051sup1.cif

Structure factors: contains datablock(s) I. DOI: 10.1107/S2414314622002656/vm4051Isup2.hkl

CCDC reference: 2157239

Additional supporting information:  crystallographic information; 3D view; checkCIF report

## Figures and Tables

**Table 1:** Hydrogen-bond geometry (Å, °)

*D*—H⋯*A*	*D*—H	H⋯*A*	*D*⋯*A*	*D*—H⋯*A*
O3—H3*A*⋯O7	0.72 (4)	2.09 (4)	2.797 (4)	167 (5)
O4—H4*A*⋯I1^i^	0.78 (5)	2.71 (5)	3.482 (3)	171 (4)
O5—H5*A*⋯I1	0.74 (5)	2.75 (5)	3.474 (3)	165 (4)
C6—H6*A*⋯O5^ii^	0.99	2.46	3.332 (4)	147
C8—H8⋯O4^ii^	0.95	2.44	3.252 (4)	143
C8—H8⋯O5^ii^	0.95	2.53	3.285 (4)	136
C9—H9⋯O3^iii^	0.95	2.51	3.404 (5)	156
C10—H10⋯O7^iii^	0.95	2.40	3.159 (5)	137
C11—H11⋯I1^iv^	0.95	3.02	3.925 (3)	161
C15—H15⋯O4	0.95	2.58	3.297 (5)	132

Symmetry codes: (i) 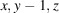 ; (ii) 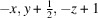 ; (iii) 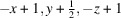 ; (iv) 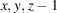 .

**Table 2:** Experimental details

Crystal data	
Chemical formula	C_12_H_19_N_2_O_5_^+^·I^−^·C_3_H_7_NO
*M*_r_	471.29
Crystal system, space group	Monoclinic, *P*2_1_
Temperature (K)	123
*a*, *b*, *c* (Å)	10.816 (2), 7.0106 (15), 13.169 (3)
β (°)	106.833 (4)
*V* (Å^3^)	955.7 (3)
*Z*	2
Radiation type	Mo *K*α
μ (mm^−1^)	1.71
Crystal size (mm)	0.29 × 0.08 × 0.03

Data collection	
Diffractometer	Bruker Kappa APEXII CCD
Absorption correction	Multi-scan (*SADABS*; Bruker, 2003 ▸)
*T*_min_, *T*_max_	0.629, 0.746
No. of measured, independent and observed [*I* > 2σ(*I*)] reflections	17430, 6072, 5626
*R*_int_	0.038
(sin θ/λ)_max_ (Å^−1^)	0.725

Refinement	
*R*[*F* ^2^ > 2σ(*F* ^2^)], *wR*(*F* ^2^), *S*	0.028, 0.060, 1.03
No. of reflections	6072
No. of parameters	242
No. of restraints	1
H-atom treatment	H atoms treated by a mixture of independent and constrained refinement
Δρ_max_, Δρ_min_ (e Å^−3^)	1.42, −0.44
Absolute structure	Refined as an inversion twin, 2815 Friedel pairs.
Absolute structure parameter	0.006 (19)

Computer programs: *APEX2* and *SAINT* (Bruker, 2003 ▸), *SHELXTL* (Sheldrick, 2008 ▸), *SHELXL2014/7* (Sheldrick, 2015 ▸) and *ORTEP-3 for Windows* (Farrugia, 2012 ▸).

## full crystallographic data

### Crystal data

**Table:**

C_12_H_19_N_2_O_5_^+^·I^−^·C_3_H_7_NO	*F*(000) = 476
*M**_r_* = 471.29	*D*_x_ = 1.638 Mg m^−^^3^
Monoclinic, *P*2_1_	Mo *K*α radiation, λ = 0.71073 Å
*a* = 10.816 (2) Å	Cell parameters from 7185 reflections
*b* = 7.0106 (15) Å	θ = 3.2–31.1°
*c* = 13.169 (3) Å	µ = 1.71 mm^−^^1^
β = 106.833 (4)°	*T* = 123 K
*V* = 955.7 (3) Å^3^	Needle, colourless
*Z* = 2	0.29 × 0.08 × 0.03 mm

### Data collection

**Table:**

Bruker Kappa APEXII CCD diffractometer	6072 independent reflections
Radiation source: sealed tube	5626 reflections with *I* > 2σ(*I*)
Detector resolution: 10.4167 pixels mm^-1^	*R*_int_ = 0.038
phi and ω scans	θ_max_ = 31.0°, θ_min_ = 3.8°
Absorption correction: multi-scan (SADABS; Bruker, 2003)	*h* = −15→15
*T*_min_ = 0.629, *T*_max_ = 0.746	*k* = −10→10
17430 measured reflections	*l* = −19→18

### Refinement

**Table:**

Refinement on *F*^2^	Hydrogen site location: mixed
Least-squares matrix: full	H atoms treated by a mixture of independent and constrained refinement
*R*[*F*^2^ > 2σ(*F*^2^)] = 0.028	*w* = 1/[σ^2^(*F*_o_^2^) + (0.0231*P*)^2^] where *P* = (*F*_o_^2^ + 2*F*_c_^2^)/3
*wR*(*F*^2^) = 0.060	(Δ/σ)_max_ = 0.001
*S* = 1.03	Δρ_max_ = 1.42 e Å^−^^3^
6072 reflections	Δρ_min_ = −0.44 e Å^−^^3^
242 parameters	Absolute structure: Refined as an inversion twin, 2815 Friedel pairs.
1 restraint	Absolute structure parameter: 0.006 (19)

### Special details

**Table:**

Geometry. All esds (except the esd in the dihedral angle between two l.s. planes) are estimated using the full covariance matrix. The cell esds are taken into account individually in the estimation of esds in distances, angles and torsion angles; correlations between esds in cell parameters are only used when they are defined by crystal symmetry. An approximate (isotropic) treatment of cell esds is used for estimating esds involving l.s. planes.
Refinement. All H atoms were positioned geometrically and refined using a riding model, with C—H = 0.98 (methyl groups), 0.99Å (methylene groups), 1.00Å (methine groups) or 0.95 Å (aryl CH) and with *U*_iso_(H) = 1.5 times *U*_eq_(C) (methyl groups) or with *U*_iso_(H) = 1.2 times *U*_eq_(C) (methylene groups, aryl CH, methine groups). Torsion angles of all methyl groups were allowed to refine. Refinement of F^2^ against ALL reflections. The weighted R-factor wR and goodness of fit S are based on F^2^, conventional R-factors R are based on F, with F set to zero for negative F^2^. The threshold expression of F^2^ > 2σ(F^2^) is used only for calculating R-factors(gt) etc. and is not relevant to the choice of reflections for refinement. R-factors based on F^2^ are statistically about twice as large as those based on F, and R- factors based on ALL data will be even larger. Refined as a two-component inversion twin.

### Fractional atomic coordinates and isotropic or equivalent isotropic displacement parameters (Å^2^)

**Table:**

	*x*	*y*	*z*	*U*_iso_*/*U*_eq_	
N1	0.1863 (3)	1.0859 (4)	0.3364 (2)	0.0151 (5)	
N2	0.1065 (3)	1.1296 (4)	0.1676 (2)	0.0179 (6)	
O1	0.3511 (2)	0.8245 (4)	0.4934 (2)	0.0146 (5)	
O2	0.3182 (3)	0.5032 (4)	0.4445 (2)	0.0184 (5)	
O3	0.4060 (3)	0.3939 (4)	0.6541 (3)	0.0222 (6)	
O4	0.2120 (3)	0.5948 (4)	0.7282 (2)	0.0197 (5)	
O5	0.0572 (2)	0.8579 (4)	0.5786 (2)	0.0169 (5)	
C1	0.3882 (3)	0.6322 (5)	0.5202 (3)	0.0150 (7)	
H1	0.4821	0.6180	0.5263	0.018*	
C2	0.3673 (3)	0.5847 (5)	0.6269 (3)	0.0156 (6)	
H2	0.4240	0.6703	0.6816	0.019*	
C3	0.2266 (3)	0.6217 (5)	0.6246 (3)	0.0137 (6)	
H3	0.1679	0.5327	0.5733	0.016*	
C4	0.1910 (3)	0.8273 (5)	0.5914 (3)	0.0129 (6)	
H4	0.2438	0.9163	0.6464	0.015*	
C5	0.2174 (3)	0.8639 (4)	0.4853 (3)	0.0123 (6)	
H5	0.1607	0.7793	0.4299	0.015*	
C6	0.1951 (3)	1.0688 (4)	0.4498 (3)	0.0139 (6)	
H6A	0.1141	1.1156	0.4618	0.017*	
H6B	0.2671	1.1487	0.4921	0.017*	
C7	0.3439 (4)	0.5229 (5)	0.3446 (3)	0.0238 (8)	
H7A	0.3086	0.4128	0.2997	0.036*	
H7B	0.4374	0.5295	0.3558	0.036*	
H7C	0.3033	0.6400	0.3097	0.036*	
C8	0.0831 (3)	1.1454 (4)	0.2617 (3)	0.0153 (7)	
H8	0.0055	1.1915	0.2729	0.018*	
C9	0.2788 (3)	1.0274 (5)	0.2893 (3)	0.0174 (7)	
H9	0.3618	0.9775	0.3244	0.021*	
C10	0.2286 (4)	1.0547 (5)	0.1843 (3)	0.0200 (7)	
H10	0.2698	1.0273	0.1313	0.024*	
C11	0.0197 (3)	1.1684 (9)	0.0656 (3)	0.0238 (7)	
H11	0.0524	1.1636	0.0060	0.029*	
C12	−0.1014 (4)	1.2099 (7)	0.0494 (3)	0.0322 (11)	
H12A	−0.1368	1.2158	0.1075	0.039*	
H12B	−0.1547	1.2344	−0.0205	0.039*	
H3A	0.426 (4)	0.391 (7)	0.711 (3)	0.014 (12)*	
H4A	0.186 (4)	0.495 (7)	0.740 (4)	0.025 (12)*	
H5A	0.051 (4)	0.940 (7)	0.612 (4)	0.018 (12)*	
I1	0.06251 (2)	1.18116 (3)	0.77975 (2)	0.02003 (6)	
N3	0.4772 (3)	0.4326 (5)	1.0319 (3)	0.0235 (7)	
O7	0.5214 (3)	0.4185 (5)	0.8731 (2)	0.0306 (7)	
C13	0.6115 (5)	0.4439 (8)	1.0952 (4)	0.0328 (10)	
H13A	0.6663	0.4637	1.0485	0.049*	
H13B	0.6363	0.3249	1.1349	0.049*	
H13C	0.6225	0.5508	1.1450	0.049*	
C14	0.3817 (4)	0.4304 (7)	1.0890 (4)	0.0341 (10)	
H14A	0.3828	0.5529	1.1250	0.051*	
H14B	0.4016	0.3275	1.1416	0.051*	
H14C	0.2960	0.4092	1.0391	0.051*	
C15	0.4445 (5)	0.4205 (6)	0.9273 (4)	0.0241 (8)	
H15	0.3550	0.4126	0.8906	0.029*	

### Atomic displacement parameters (Å^2^)

**Table:**

	*U*^11^	*U*^22^	*U*^33^	*U*^12^	*U*^13^	*U*^23^
N1	0.0161 (13)	0.0129 (12)	0.0185 (14)	−0.0016 (10)	0.0086 (11)	−0.0002 (11)
N2	0.0226 (14)	0.0170 (14)	0.0156 (13)	−0.0010 (10)	0.0079 (11)	0.0006 (10)
O1	0.0115 (12)	0.0128 (12)	0.0203 (13)	−0.0008 (9)	0.0061 (10)	0.0012 (10)
O2	0.0274 (14)	0.0139 (12)	0.0178 (13)	−0.0039 (10)	0.0125 (11)	−0.0033 (11)
O3	0.0272 (15)	0.0191 (12)	0.0205 (14)	0.0079 (10)	0.0072 (12)	0.0051 (11)
O4	0.0252 (13)	0.0200 (12)	0.0170 (12)	0.0001 (10)	0.0109 (10)	0.0032 (10)
O5	0.0146 (12)	0.0199 (12)	0.0185 (12)	0.0001 (9)	0.0081 (10)	−0.0027 (10)
C1	0.0138 (14)	0.0141 (17)	0.0181 (15)	0.0027 (10)	0.0064 (12)	0.0005 (11)
C2	0.0163 (15)	0.0147 (15)	0.0158 (15)	0.0024 (11)	0.0049 (12)	0.0002 (12)
C3	0.0156 (15)	0.0140 (13)	0.0132 (14)	−0.0010 (11)	0.0065 (12)	−0.0021 (11)
C4	0.0130 (14)	0.0130 (14)	0.0135 (15)	0.0004 (11)	0.0052 (12)	−0.0024 (13)
C5	0.0108 (14)	0.0131 (13)	0.0137 (15)	−0.0002 (10)	0.0047 (12)	−0.0014 (12)
C6	0.0170 (15)	0.0132 (13)	0.0135 (15)	0.0008 (11)	0.0075 (12)	−0.0004 (12)
C7	0.038 (2)	0.0196 (17)	0.0184 (17)	−0.0027 (15)	0.0156 (16)	−0.0031 (14)
C8	0.0205 (14)	0.011 (2)	0.0169 (14)	−0.0008 (10)	0.0085 (11)	0.0021 (11)
C9	0.0175 (16)	0.0153 (15)	0.0237 (18)	−0.0017 (12)	0.0129 (14)	−0.0021 (13)
C10	0.0234 (17)	0.0175 (15)	0.0241 (18)	−0.0043 (13)	0.0149 (15)	−0.0032 (14)
C11	0.0362 (17)	0.0203 (18)	0.0148 (13)	0.001 (2)	0.0072 (12)	0.005 (2)
C12	0.042 (2)	0.030 (3)	0.0215 (16)	0.0067 (18)	0.0031 (15)	0.0061 (18)
I1	0.02599 (10)	0.01685 (9)	0.01752 (9)	0.00241 (13)	0.00673 (7)	0.00025 (13)
N3	0.0222 (16)	0.0241 (16)	0.0226 (17)	−0.0012 (13)	0.0040 (13)	0.0042 (14)
O7	0.0305 (16)	0.0392 (17)	0.0239 (15)	0.0009 (13)	0.0108 (13)	0.0044 (13)
C13	0.028 (2)	0.041 (2)	0.024 (2)	0.003 (2)	−0.0027 (18)	0.0025 (19)
C14	0.033 (2)	0.042 (2)	0.031 (2)	−0.0034 (19)	0.0139 (19)	0.005 (2)
C15	0.022 (2)	0.0260 (18)	0.022 (2)	−0.0009 (17)	0.0021 (18)	0.0034 (17)

### Geometric parameters (Å, º)

**Table:**

N1—C8	1.324 (4)	C5—H5	1.0000
N1—C9	1.383 (4)	C6—H6A	0.9900
N1—C6	1.472 (4)	C6—H6B	0.9900
N2—C8	1.339 (4)	C7—H7A	0.9800
N2—C10	1.379 (5)	C7—H7B	0.9800
N2—C11	1.424 (4)	C7—H7C	0.9800
O1—C1	1.421 (4)	C8—H8	0.9500
O1—C5	1.446 (4)	C9—C10	1.345 (5)
O2—C1	1.396 (4)	C9—H9	0.9500
O2—C7	1.428 (5)	C10—H10	0.9500
O3—C2	1.416 (4)	C11—C12	1.298 (6)
O3—H3A	0.72 (4)	C11—H11	0.9500
O4—C3	1.430 (4)	C12—H12A	0.9500
O4—H4A	0.78 (5)	C12—H12B	0.9500
O5—C4	1.424 (4)	N3—C15	1.322 (6)
O5—H5A	0.74 (5)	N3—C14	1.442 (6)
C1—C2	1.523 (5)	N3—C13	1.453 (5)
C1—H1	1.0000	O7—C15	1.243 (5)
C2—C3	1.536 (5)	C13—H13A	0.9800
C2—H2	1.0000	C13—H13B	0.9800
C3—C4	1.523 (5)	C13—H13C	0.9800
C3—H3	1.0000	C14—H14A	0.9800
C4—C5	1.527 (5)	C14—H14B	0.9800
C4—H4	1.0000	C14—H14C	0.9800
C5—C6	1.509 (4)	C15—H15	0.9500
			
C8—N1—C9	108.9 (3)	C5—C6—H6A	109.6
C8—N1—C6	124.9 (3)	N1—C6—H6B	109.6
C9—N1—C6	126.0 (3)	C5—C6—H6B	109.6
C8—N2—C10	108.2 (3)	H6A—C6—H6B	108.1
C8—N2—C11	127.4 (3)	O2—C7—H7A	109.5
C10—N2—C11	124.2 (3)	O2—C7—H7B	109.5
C1—O1—C5	113.8 (3)	H7A—C7—H7B	109.5
C1—O2—C7	112.6 (3)	O2—C7—H7C	109.5
C2—O3—H3A	105 (4)	H7A—C7—H7C	109.5
C3—O4—H4A	117 (4)	H7B—C7—H7C	109.5
C4—O5—H5A	108 (3)	N1—C8—N2	108.5 (3)
O2—C1—O1	112.4 (3)	N1—C8—H8	125.8
O2—C1—C2	108.8 (3)	N2—C8—H8	125.8
O1—C1—C2	109.3 (3)	C10—C9—N1	106.9 (3)
O2—C1—H1	108.8	C10—C9—H9	126.6
O1—C1—H1	108.8	N1—C9—H9	126.6
C2—C1—H1	108.8	C9—C10—N2	107.5 (3)
O3—C2—C1	109.2 (3)	C9—C10—H10	126.2
O3—C2—C3	112.5 (3)	N2—C10—H10	126.2
C1—C2—C3	110.9 (3)	C12—C11—N2	123.8 (3)
O3—C2—H2	108.0	C12—C11—H11	118.1
C1—C2—H2	108.0	N2—C11—H11	118.1
C3—C2—H2	108.0	C11—C12—H12A	120.0
O4—C3—C4	108.1 (3)	C11—C12—H12B	120.0
O4—C3—C2	110.0 (3)	H12A—C12—H12B	120.0
C4—C3—C2	109.4 (3)	C15—N3—C14	121.8 (4)
O4—C3—H3	109.8	C15—N3—C13	121.5 (4)
C4—C3—H3	109.8	C14—N3—C13	116.7 (4)
C2—C3—H3	109.8	N3—C13—H13A	109.5
O5—C4—C3	109.9 (3)	N3—C13—H13B	109.5
O5—C4—C5	108.6 (3)	H13A—C13—H13B	109.5
C3—C4—C5	108.9 (3)	N3—C13—H13C	109.5
O5—C4—H4	109.8	H13A—C13—H13C	109.5
C3—C4—H4	109.8	H13B—C13—H13C	109.5
C5—C4—H4	109.8	N3—C14—H14A	109.5
O1—C5—C6	105.7 (3)	N3—C14—H14B	109.5
O1—C5—C4	110.4 (3)	H14A—C14—H14B	109.5
C6—C5—C4	112.8 (3)	N3—C14—H14C	109.5
O1—C5—H5	109.3	H14A—C14—H14C	109.5
C6—C5—H5	109.3	H14B—C14—H14C	109.5
C4—C5—H5	109.3	O7—C15—N3	125.3 (5)
N1—C6—C5	110.4 (3)	O7—C15—H15	117.4
N1—C6—H6A	109.6	N3—C15—H15	117.4
			
C7—O2—C1—O1	65.7 (4)	O5—C4—C5—C6	64.9 (3)
C7—O2—C1—C2	−173.1 (3)	C3—C4—C5—C6	−175.4 (3)
C5—O1—C1—O2	61.2 (4)	C8—N1—C6—C5	117.5 (3)
C5—O1—C1—C2	−59.7 (3)	C9—N1—C6—C5	−56.3 (4)
O2—C1—C2—O3	57.7 (3)	O1—C5—C6—N1	74.9 (3)
O1—C1—C2—O3	−179.2 (3)	C4—C5—C6—N1	−164.3 (3)
O2—C1—C2—C3	−66.8 (3)	C9—N1—C8—N2	−0.8 (4)
O1—C1—C2—C3	56.3 (3)	C6—N1—C8—N2	−175.6 (3)
O3—C2—C3—O4	63.2 (4)	C10—N2—C8—N1	0.9 (4)
C1—C2—C3—O4	−174.2 (3)	C11—N2—C8—N1	176.6 (4)
O3—C2—C3—C4	−178.2 (3)	C8—N1—C9—C10	0.5 (4)
C1—C2—C3—C4	−55.6 (3)	C6—N1—C9—C10	175.1 (3)
O4—C3—C4—O5	−66.2 (3)	N1—C9—C10—N2	0.1 (4)
C2—C3—C4—O5	174.1 (3)	C8—N2—C10—C9	−0.6 (4)
O4—C3—C4—C5	175.0 (3)	C11—N2—C10—C9	−176.5 (4)
C2—C3—C4—C5	55.3 (3)	C8—N2—C11—C12	−6.4 (8)
C1—O1—C5—C6	−176.5 (3)	C10—N2—C11—C12	168.6 (5)
C1—O1—C5—C4	61.2 (3)	C14—N3—C15—O7	−178.8 (4)
O5—C4—C5—O1	−177.0 (3)	C13—N3—C15—O7	−0.4 (7)
C3—C4—C5—O1	−57.3 (3)		

### Hydrogen-bond geometry (Å, º)

**Table:**

*D*—H···*A*	*D*—H	H···*A*	*D*···*A*	*D*—H···*A*
O3—H3*A*···O7	0.72 (4)	2.09 (4)	2.797 (4)	167 (5)
O4—H4*A*···I1^i^	0.78 (5)	2.71 (5)	3.482 (3)	171 (4)
O5—H5*A*···I1	0.74 (5)	2.75 (5)	3.474 (3)	165 (4)
C6—H6*A*···O5^ii^	0.99	2.46	3.332 (4)	147
C8—H8···O4^ii^	0.95	2.44	3.252 (4)	143
C8—H8···O5^ii^	0.95	2.53	3.285 (4)	136
C9—H9···O3^iii^	0.95	2.51	3.404 (5)	156
C10—H10···O7^iii^	0.95	2.40	3.159 (5)	137
C11—H11···I1^iv^	0.95	3.02	3.925 (3)	161
C15—H15···O4	0.95	2.58	3.297 (5)	132

Symmetry codes: (i) *x*, *y*−1, *z*; (ii) −*x*, *y*+1/2, −*z*+1; (iii) −*x*+1, *y*+1/2, −*z*+1; (iv) *x*, *y*, *z*−1.
